# Synthesis and cytotoxicity of novel cyanochalcones: induction of cell cycle arrest, apoptosis, and autophagy in HEP2 and MCF-7 cells

**DOI:** 10.1007/s00210-025-04940-z

**Published:** 2026-01-15

**Authors:** Nada S. Ibrahim, Amr Ahmed WalyEldeen, Sherif Abdelaziz Ibrahim, Ismail A. Abdelhamid, Heba K. A. Elhakim

**Affiliations:** 1 Department of Chemistry (Biochemistry Division), Faculty of Science, Cairo University, Giza, 12613 Egypt; 2 Department of Zoology, Faculty of Science, Cairo University, Giza, 12613 Egypt; 3 Department of Chemistry, Faculty of Science, Cairo University, Giza, 12613 Egypt

**Keywords:** Cyanochalcones, Ferroptosis, Apoptosis, Autophagy, Molecular docking

## Abstract

Developing new chemotherapeutic agents is essential for improving cancer treatment and overcoming resistance. In this study, four novel synthetic cyanochalcone derivatives were designed, synthesized, and structurally characterized using various spectroscopic techniques. Their anticancer potential was evaluated via an MTT cytotoxicity assay against three human cancer cell lines and one normal cell line. Among the tested compounds, **5b** exhibited the highest cytotoxic activity against laryngeal carcinoma HEP2 cells (IC_50_ = 23.93 ± 1.3 µM), whereas **5c** demonstrated potent cytotoxicity against breast cancer MCF-7 cells (IC_50_ = 39.08 ± 3.4 µM). Mechanistically, treatment of HEP2 cells with compound **5b** significantly downregulated cyclin-dependent kinase (*CDK*)*4* and *CDK6*, key regulators of the cell cycle, while modulating ferroptosis-associated genes by suppressing transferrin (*TF*) and upregulating heme oxygenase-1 (*HO-1*). In contrast, compound **5c** induced apoptosis in MCF-7 cells by upregulating *TF* while downregulating autophagy-related 3 (*ATG3*). Flow cytometric analysis indicated that both compounds promoted *ATG3*-independent autophagy and cell cycle arrest at G0/G1 (in HEP2) and G2/M (in MCF-7). Furthermore, these chalcone derivatives effectively induced P53-independent apoptosis, as evidenced by a significant increase in both early and late apoptotic phases. Molecular docking studies demonstrated a strong binding affinity of compound **5b** toward *CDK4* and *CDK6*, supporting its role in cell cycle inhibition. Overall, these findings suggest that the synthetic chalcone derivatives **5b** and **5c** possess promising anticancer activity, modulating key pathways involved in cell cycle regulation, apoptosis, ferroptosis, and autophagy, and emerge as strong candidates for further investigation in the development of new therapeutic agents.

**Supplementary Information:**

The online version contains supplementary material available at 10.1007/s00210-025-04940-z.

## Introduction

Cancer continues to be the world’s most significant cause of mortality, and the number of deaths from it rises every day (Isyaka et al. 2025). Nearly 10 million people died from cancer worldwide in 2020 alone. Cancer is marked by the unregulated growth of cells within the body, stemming from cellular control disturbances and cell cycle disruption (Silva et al. 2025).

Each year, approximately 287,850 new cases of breast cancer are diagnosed, leading to more than 43,250 fatalities (Dabhade et al. 2025). In women, it is the leading cause of cancer-related fatalities, representing 31% of all cancer cases and 15% of cancer deaths. The primary reason for female mortality worldwide is the onset of metastatic breast cancer (Dabhade et al. 2025).

Laryngeal cancer, also known as laryngeal carcinoma, is a type of cancer that targets the tissues of the larynx (Fahmy et al. 2025). In the upper aerodigestive tract, it is the second most common type of cancer. Laryngeal cancer is a prevalent malignancy that kills about 83,000 people worldwide every year (Zhu et al. 2024).

Olefins of biological significance are often produced via Knoevenagel condensation (Mohamed et al. 2020; Sroor et al. 2020; Sroor et al. 2019). Chalcones with a keto-ethylenic linkage between the rings (A and B) are interesting secondary metabolites with anti-inflammatory (Bandgar et al. 2009; Bekhit and Abdel-Aziem 2004), antioxidant (Bandgar et al. 2009; Shenvi et al. 2013), antiplatelet (Lin et al. 2001), antimalarial (Li et al. 1995), antibacterial (Asiri and Khan 2011; Mohamed et al. 2012), analgesic (Heidari et al. 2009), and anticancer properties (Mohamed et al. 2014; Mohamed et al. 2012; Shenvi et al. 2013). The bioactivity of chalcones is attributed to their reactive a,ß-unsaturated enone moiety. Mohamed et al. reported the promising anticancer effect of an α-cyanochalcone derivative against HCT-116 colon carcinoma via induction of extrinsic and intrinsic apoptotic pathways (Mohamed et al. 2023). Kumar et al. reported the most potent and selective effect of α-cyano bis(indolyl)chalcone derivative against A549 lung cancer cells (Kumar et al. 2014). Hence, various publications have been published on new derivatives, including structural changes to the basic enone unit. In continuation of our interest in the synthesis of bioactive heterocycles (Elgamal et al. 2025; Mohamed et al. 2025; Ragheb et al. 2025a, b; Ragheb et al. 2025a, b; Salem et al. 2025; WalyEldeen et al. 2022; WalyEldeen et al. 2023), this study aims to synthesize and characterize novel cyanochalcone derivatives and investigate their cytotoxic mechanisms in breast and laryngeal carcinoma cells.

## Results and discussion

### Chemistry

**Scheme 1:**
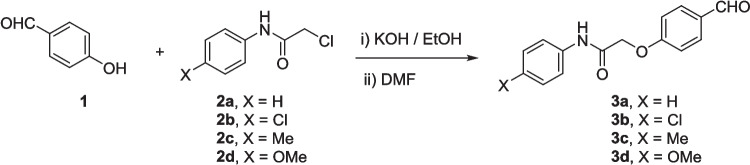
Synthesis of 2-(4-formylphenoxy)-*N*-arylacetamide precursors **3a–d**

**Scheme 2:**
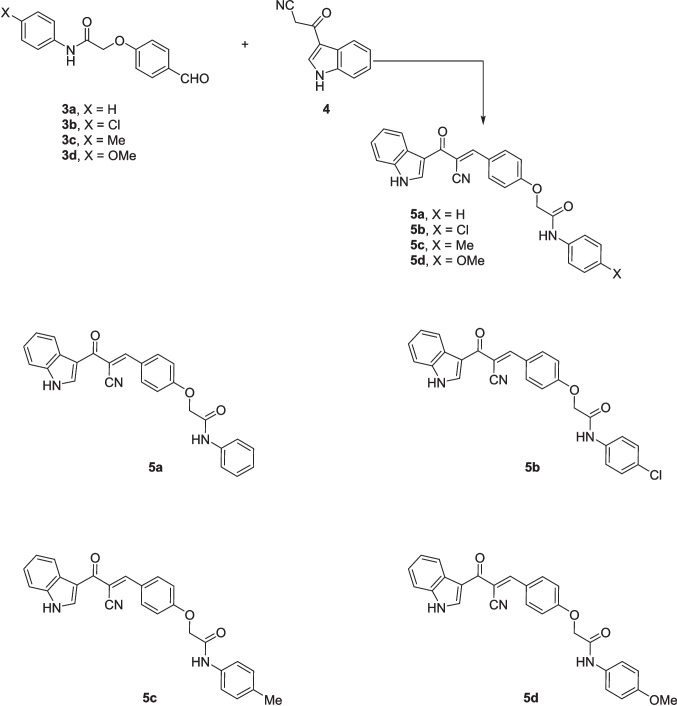
Synthesis of 2-(4-(2-cyano-3-(1*H*-indol-3-yl)−3-oxoprop-1-en-1-yl)phenoxy)-*N*-arylacetamides **5a–d**

The precursors 2-(4-formylphenoxy)-*N*-arylacetamides **3a–d** were obtained as we have previously reported (Abdelwahab et al. 2023; Omar et al. 2021), via combining 4-hydroxybenzaldehyde **1** with the corresponding 2-chloro-*N*-arylacetamide **2a–d** in the presence of KOH (Scheme 1). Subsequently, the cyanochalcones: 2-(4-(2-cyano-3-(1*H*-indol-3-yl)−3-oxoprop-1-en-1-yl)phenoxy)-*N*-arylacetamides **5a–d** were produced by the Claisen-Schmidt condensation reaction of 2-(4-formylphenoxy)-*N*-arylacetamide precursors **3a–d** with 3-(1*H*-indol-3-yl)−3-oxopropanenitrile **4** in ethanol in the presence of piperidine as a basic catalyst (Scheme 2).

### Biology

#### Screening of the cytotoxic impact of different chalcone derivatives on cancer cell lines

**Fig. 1:**
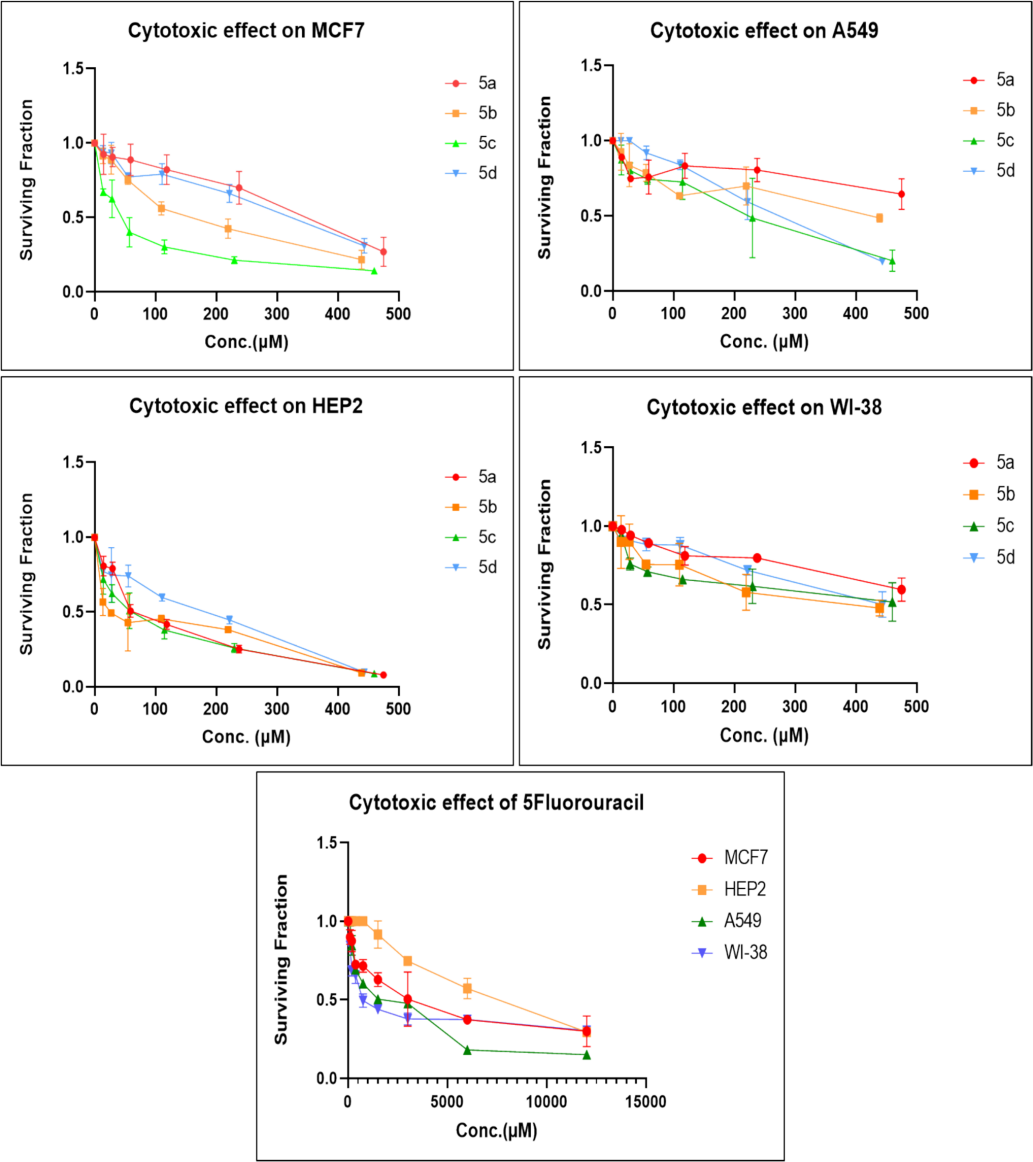
Cytotoxic effects of chalcone derivatives (**5a–d**) on cancer cell lines (A549, MCF-7, and HEP2) and WI-38 normal cell line, as evaluated by the MTT assay. The graphs display the dose-dependent reduction in cell viability in response to increasing concentrations of each compound. Data are presented as mean ± SD (*n* ≥ 3). The solid lines represent nonlinear regression fits used for IC₅₀ determination

The cytotoxic potential of the synthesized chalcone derivatives (**5a–d**) was assessed using the MTT assay across a panel of cancer cell lines, including lung carcinoma (A549), breast cancer (MCF-7), and laryngeal carcinoma (HEP2), along with a normal fibroblast cell line (WI-38) to evaluate drug selectivity. IC_50_ values and selectivity index (SI) were calculated to determine the potency and selectivity of each compound (Table 1 and Fig. 1). SI = IC_50_ of normal cell/IC_50_ of cancer cell. Results indicated that the chalcone derivatives exhibited notable selective cytotoxicity, especially against MCF-7 and HEP2 cells. Among them, compound **5c** showed a potent effect against MCF-7 cells with an IC_50_ of 39.08 ± 3.4 µM, relative to the reference drug 5-Fluorouracil (5-FU). In addition, compound **5c** had the greatest selective effect toward MCF-7 with SI > 11 compared to the other derivatives, which had SI > 1. Similarly, compound **5b** demonstrated potent activity against HEP2 cells with an IC_50_ of 23.93 ± 1.3 µM, which was markedly more effective than 5-FU and also surpassed the cytotoxic performance of two previously reported chalcone analogs: N-(4-Methoxyphenyl)−2-(4-(3-oxo-3-(thiophen-2-yl)prop-1-en-1-yl)phenoxy)acetamide (IC_50_ = 30.5 µM) and N-Phenyl-2-(4-(3-(thiophen-2-yl)acryloyl)phenoxy)acetamide (IC_50_ = 42.7 µM) (Ibrahim et al. 2024). Also, the highest selectivity against HEP2 was shown for compound **5b** with SI > 15 compared to **5a**, **5c**, and **5d**, which had SI > 12, > 7, and > 1, respectively.

**Table 1:** The IC_50_ ± standard deviation (SD) values of the novel chalcones and the positive control 5-FU on human cancer cell lines A549, MCF7, HEP2, and normal cell line (WI-38)

Cell lines	IC_50_ Values (µM)				
	**5-FU**	**5a**	**5b**	**5c**	**5d**
**A549**	1458.51 ± 1.6	IA	432.07 ± 2.8	225.98 ± 26.4	265.76 ± 11.5
**MCF-7**	3017.15 ± 17.0	351.99 ± 11	167.73 ± 4.0	39.08 ± 3.4	324.39 ± 6.0
**HEP2**	7466.74 ± 6.5	38.72 ± 4.3	23.93 ± 1.3	64.83 ± 11.8	351.66 ± 2.7
**Normal (WI-38)**	661.82 ± 4.2	> 475.00	376.11 ± 11.4	460.56 ± 12.3	452.50 ± 8.2

*IA* inactive

**Scheme 3:**
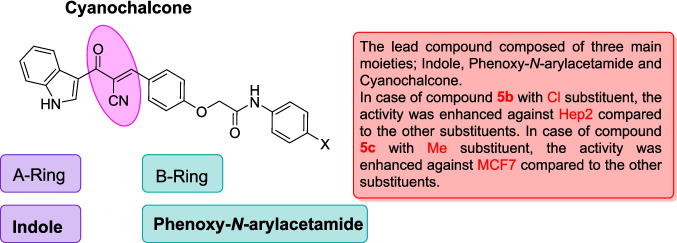
The design strategy of the prepared compounds

These findings suggest that **5b** holds a strong potential as a therapeutic candidate for targeting laryngeal cancer. Compound **5a** also showed promising activity against HEP2 cells (IC_50_ = 38.72 ± 4.3 µM) compared to 5-FU. In contrast, the chalcone derivatives displayed mild activity against the A549 lung cancer cell line, with compound **5c** showing the least potency (IC_50_ = 225.98 ± 26.4 µM) with SI > 2. Despite this reduced effect in A549 cells, compounds **5b–d** still outperformed 5-FU, indicating their superior cytotoxic profile across multiple cancer types. Based on the structure–activity relationship, it was noticed that the derivative **5b** bearing a chloro substituent had the highest cytotoxic effect against HEP2, followed by the unsubstituted derivative **5a**, then the methyl-substituted **5c,** and finally the methoxy-substituted ** 5d,** with the order of Cl > unsubstituted > methyl > methoxy. Therefore, the electron-withdrawing ability and lower steric hindrance of chloro compared with other substituents enhanced the cytotoxic effect of **5b** against HEP2. The methyl substituent (electron-donating) in derivative **5c** strengthened the cytotoxic effect against MCF-7 compared to the others (Scheme 3).

Notably, the chalcone derivatives exhibited minimal cytotoxicity toward the normal WI-38 fibroblast cell line, with no significant toxicity observed at the tested concentrations. This selective cytotoxic effect against cancer cells, while sparing normal cells, highlights their potential as therapeutically safe anticancer agents. In summary, the data indicate that compound **5b** is the most promising candidate against HEP2 cells, while compound **5c** shows the greatest efficacy against MCF-7 cells. Due to their potent cytotoxic activity and selectivity, we have focused on these two compounds for the subsequent experiments. These results are consistent with previous studies highlighting the anticancer properties of chalcones, which have been shown to induce cell cycle arrest and apoptosis via multiple molecular mechanisms. The observed selective cytotoxicity is particularly significant, as it addresses a key challenge in cancer therapy—minimizing off-target toxicity, a common drawback of conventional chemotherapeutic agents (Buyukgolcigezli et al. 2025).

#### Chalcones 5b and 5c induce autophagy, cell cycle arrest, and apoptosis in HEP2 and MCF7 cells

**Fig. 2:**
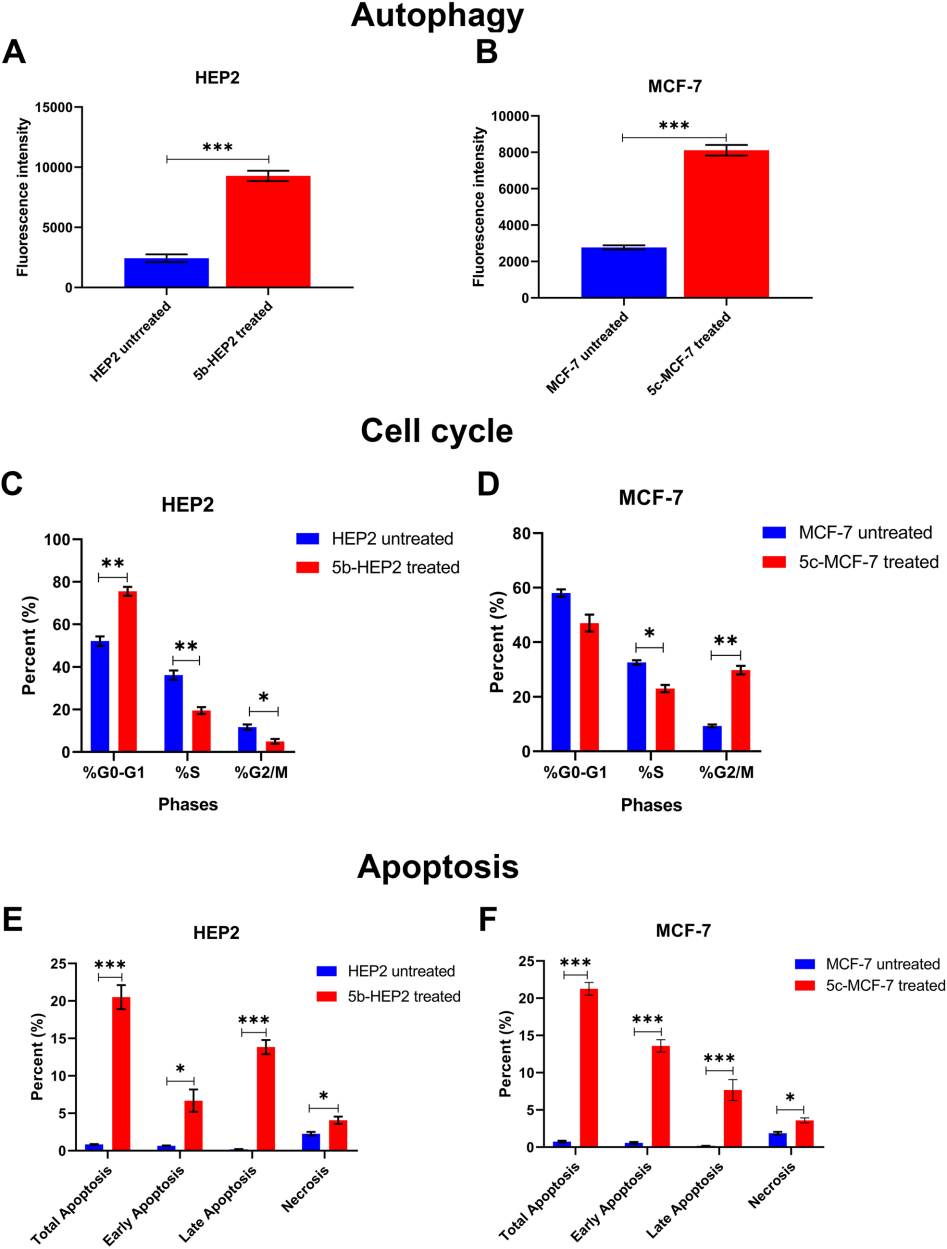
Effects of chalcone derivatives 5b and 5c on autophagy, cell cycle progression, and apoptosis in HEP2 and MCF-7 cells. HEP2 cells were treated with compound 5b (23.93 µM, IC₅₀) and MCF-7 cells were treated with compound 5c (39.08 µM, IC₅₀) for 48 h; untreated cells served as negative controls. **A** and **B** Quantification of CYTO-ID® mean fluorescence intensity (MFI) as a measure of autophagy in untreated and treated cells. **C** and **D** Quantification of the percentages of cells in G0/G1, S, and G2/M phases. **E** and **F** Quantification of apoptotic and necrotic cell populations. Total apoptosis corresponds to the sum of early and late apoptotic cells. Data are presented as mean ± SEM (*n* ≥ 3). Statistical significance between treated and untreated groups was evaluated using an unpaired two-tailed Student’s *t*-test; *P < 0.05, **P < 0.01, ***P < 0.001 vs. the corresponding untreated control

Flow cytometry analysis demonstrated a significant increase in autophagic activity in both HEP2 and MCF-7 cells following 48 h of treatment. In HEP2 cells, treatment with the IC_50_ value of compound **5b** caused a substantial increase in autophagic activity, with mean fluorescence intensity rising from 2429 ± 328 in untreated cells to 9278 ± 418 in treated cells (*P* < 0.001). Similarly, MCF-7 cells treated with the IC_50_ of compound **5c** showed a marked elevation in autophagy, with fluorescence intensity increasing from 2772 ± 129 to 8111 ± 286 (*P* < 0.001). These findings suggest that **5b** and **5c** promote autophagy, which may contribute to their cytotoxic effects on HEP2 and MCF-7 cells, respectively (Fig. 2A–B). Based on qPCR data, we propose that this autophagic response occurs independently of the ATG3 and LC3B pathways (Chang et al. 2013). It is worth mentioning that autophagy has a dual role in cancer cells. It can either make cancer cells chemoresistant or chemosensitive (Angela et al. 2022; Kwantwi 2024; Wang et al. 2024). So, herein our results demonstrate that autophagy activation enhances the cancer sensitivity towards drugs.

Cell cycle analysis confirmed distinct arrest patterns induced by compounds **5b** and **5c** (Fig. 2C–D). In HEP2 cells, 48-h treatment with the IC_50_ of compound **5b** resulted in a pronounced G0/G1 arrest, with the G0/G1 population increasing from 52.12 ± 2.19% in untreated cells to 75.53 ± 2.09% in treated cells (*P* < 0.01). This shift was accompanied by a reduction in the S phase from 36.20 ± 2.15% to 19.51 ± 1.61% (*P* < 0.01), and a decrease in the G2/M population from 11.68 ± 1.25% to 4.96 ± 1.12% (*P* < 0.05), confirming strong G1-phase blockade. This finding aligns with qPCR results showing significant downregulation of CDK4 and CDK6. In addition, this result is consistent with our previous studies on related chalcone derivatives, which also induced G0/G1 arrest in HeLa cells (Ibrahim et al. 2025). In contrast, MCF-7 cells treated with the IC_50_ of compound **5c** exhibited a clear G2/M arrest rather than a G0/G1 arrest. Compound **5c** increased the G2/M fraction from 9.33 ± 0.53% in untreated cells to 29.80 ± 1.59% in treated cells (*P* < 0.01). Correspondingly, the G0/G1 population decreased from 58.04 ± 1.31% to 47.00 ± 3.12%, and the S phase declined from 32.64 ± 0.78% to 23.03 ± 1.35% (*P* < 0.05). These results indicate that **5b** and **5c** inhibit cancer cell proliferation by inducing cell cycle arrest at the G0/G1 and G2/M phases, respectively.

Furthermore, flow cytometry analysis confirmed that both compounds induced apoptosis in their respective cell lines (Fig. 2E–F). In HEP2 cells, treatment with the IC_50_ of compound **5b** resulted in a marked and statistically significant increase in total apoptosis, rising from 0.84 ± 0.05% in untreated cells to 20.52 ± 1.61% in treated cells (*P* < 0.001). Early apoptosis increased from 0.66 ± 0.05% to 6.68 ± 1.48% (*P* < 0.05), while late apoptosis increased sharply from 0.18 ± 0.04% to 13.83 ± 0.96% (*P* < 0.001). Necrosis also showed a modest but significant elevation from 2.26 ± 0.24% to 4.06 ± 0.49% (*P* < 0.05). These results indicate that compound **5b** robustly induces apoptosis in HEP2 cells. Similarly, in MCF-7 cells, treatment with the IC_50_ of compound **5c** significantly increased apoptotic cell death. Total apoptosis rose from 0.74 ± 0.12% to 21.26 ± 0.85% (*P* < 0.001). Early apoptosis was strongly elevated from 0.58 ± 0.13% to 13.59 ± 0.82% (*P* < 0.001), and late apoptosis increased from 0.17 ± 0.01% to 7.67 ± 1.41% (*P* < 0.001). A smaller but significant increase in necrosis was also observed (from 1.87 ± 0.19% to 3.59 ± 0.35%, *P* < 0.05). This pattern suggests that compound **5c** is primarily a trigger of apoptosis in MCF-7 cells. Based on qPCR analysis of apoptosis-related genes, the observed apoptosis appears to be P53-independent. This finding has therapeutic significance, as P53-independent pathways may offer effective strategies against tumors with P53 mutations, which often confer resistance to conventional treatments (McNamee and Brodsky 2009). The significance of this finding lies in its therapeutic implications; many tumors harbor P53 mutations that confer resistance to conventional therapies (Buyukgolcigezli et al. 2025).

#### Modulation of cell cycle, apoptosis, ferroptosis, and autophagy-related gene expression by chalcone derivatives 5b and 5c in HEP2 and MCF-7 cells

**Fig. 3:**
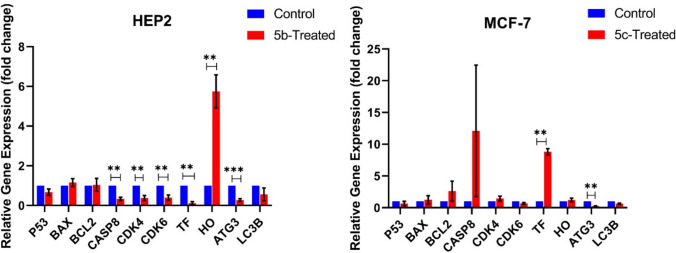
Relative gene expression in MCF-7 and HEP2 cells following chalcone treatment. The mRNA expression levels of various genes involved in apoptosis (*P53, BAX, BCL2,* and *CASP8*), cell cycle regulation (*CDK4* and *CDK6*), ferroptosis (*TF* and *HO*-*1*), and autophagy (*ATG3, LC3B*) were analyzed using qPCR in HEP2 (left) and MCF-7 (right) cell lines. The bar graphs depict the fold change in gene expression relative to the control group. Data are presented as mean ± SEM (*n* ≥ 3). Significant differences between control and treated groups are indicated by asterisks: **P < 0.01, ***P < 0.001 as determined by Student’s *t*-test

The expression levels of key regulatory genes involved in apoptosis (*P53*, *BAX*, *BCL2*, *caspase-8*), cell cycle control (*CDK4*, *CDK6*), ferroptosis (*TF*, *HO-1*), and autophagy (*ATG3*, *LC3B*) were measured in MCF-7 and HEP2 cancer cell lines following 48 h of treatment with the IC_50_ values of compounds **5c** and **5b**, respectively (Fig. 3). CDK4 and CDK6, cyclin-dependent kinases, play crucial roles in regulating the G1-to-S phase transition of the cell cycle, promoting cell proliferation. These kinases’ aberrant activation and dysregulated expression are commonly associated with tumor progression, making them important targets for anticancer therapy (Mohamed et al. 2018).

Heme oxygenase-1 (HO-1) is a vital enzyme that breaks down heme into ferrous iron, biliverdin, and carbon monoxide, thereby influencing intracellular iron homeostasis (Neubauer and Sunderram 2012). This process is intricately linked to the production of reactive oxygen species (ROS) through iron-catalyzed reactions. The accumulation of ROS induces lipid peroxidation, ultimately triggering ferroptosis, a unique form of regulated cell death characterized by iron-dependent oxidative damage to the cellular membrane (Dixon and Stockwell 2014). Furthermore, glutathione peroxidase 4 (GPX4) inactivation leads to phospholipid hydroperoxides (PLOOH) accumulation, causing irreversible cell membrane damage and ferroptosis. Importantly, ferroptosis has emerged as a promising therapeutic target in cancer treatment, as it has been shown to reverse resistance to conventional therapies (Manz et al. 2016).

Autophagy is a lysosome-mediated degradation process that plays a dual role in cancer, functioning as both a survival mechanism and a therapeutic resistance factor. By degrading cytoplasmic constituents, autophagy enables tumor cells to withstand metabolic stress and evade therapy-induced apoptosis. Consequently, suppressing key autophagy-related genes may enhance tumor sensitivity to treatment, offering a potential strategy to overcome chemo-resistance in cancer therapy (Bhutia et al. 2013). Herein, in MCF-7 cells, *TF* expression was significantly upregulated by approximately 8.8-fold (*P* < 0.01) in the **5c**-treated group, whereas *ATG3* expression was downregulated considerably to 0.23-fold (*P* < 0.001) compared to the untreated control, suggesting a potential inhibition of autophagy. Our findings are supported by Huang et al. and Levine and Kroemer’s results, as the *ATG3* gene knockdown led to the suppression of the growth and invasion of cancer cells (Huang et al. 2019; Levine and Kroemer 2019).

In the HEP2 cell line, treatment with **5b** resulted in several significant changes in gene expression. *Casp8* expression was significantly downregulated to 0.33-fold (*P* < 0.01). Furthermore, *CDK4* and *CDK6* were downregulated considerably with fold changes of 0.38 (*P* < 0.01) and 0.40 (*P* < 0.01), respectively, suggesting an impact on cell cycle progression. This result coincided with a previously reported cyanochalcone which caused *CDK4* downregulation in HCT-116 cells (Mohamed et al. 2023). The ferroptosis marker *TF* was also significantly downregulated to 0.12-fold (*P* < 0.01). *ATG3* expression showed downregulation of 0.28-fold (*P* < 0.001), and *HO-1* expression was upregulated by 5.75-fold compared to the control untreated group (*P* < 0.01).

On the other hand, the expression of *P53* (0.67-fold), *BAX* (1.16-fold), *BCL2* (1.04-fold), and *LC3B* (0.56-fold) did not show statistically significant differences when compared to the untreated control (*P* > 0.05). These findings indicated that **5c** and **5b** significantly influenced specific gene expressions in MCF-7 and HEP2 cells. In MCF-7 cells, **5c** particularly affected pathways related to ferroptosis (*TF*) and autophagy (*ATG3*), showing significant upregulation and downregulation, respectively. In HEP2 cells, **5b** had a substantial impact on apoptosis (*Casp8*), cell cycle regulation (*CDK4* and *CDK6*), ferroptosis (*HO-1* and *TF*), and autophagy (*ATG3*), with significant changes in the expression of these genes (*P* < 0.05). The differential regulation of these specific pathways may contribute to the potential therapeutic effects of **5c** and **5b** in cancer treatment.

#### Molecular docking

##### Chalcone 5b can bind to CDK4 and CDK6

**Fig. 4:**
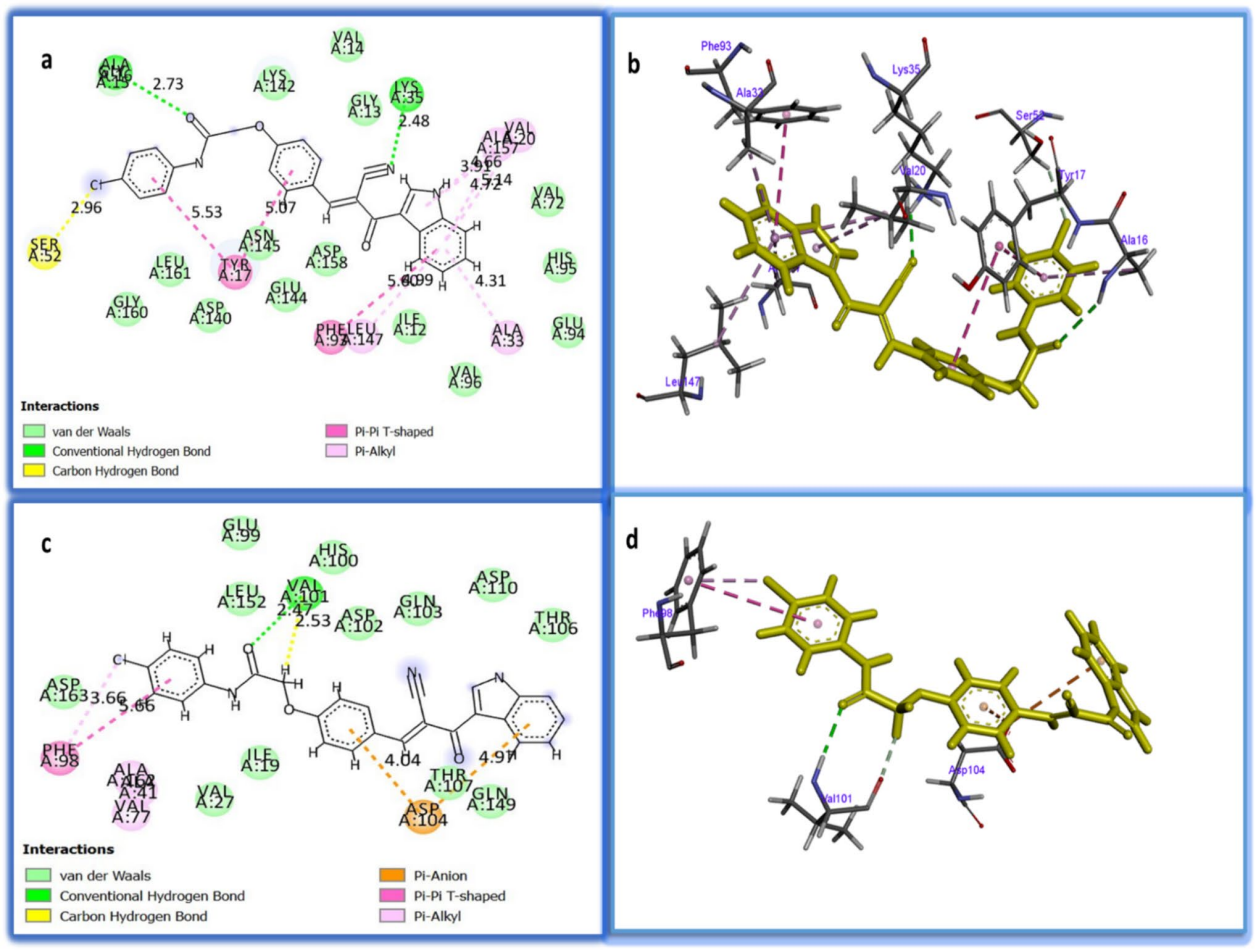
Two and three-dimensional representations of the molecular interaction between **a** and **b** compound **5b** and CDK4, **c** and **d** compound **5b** and CDK6

**Fig. 5:**
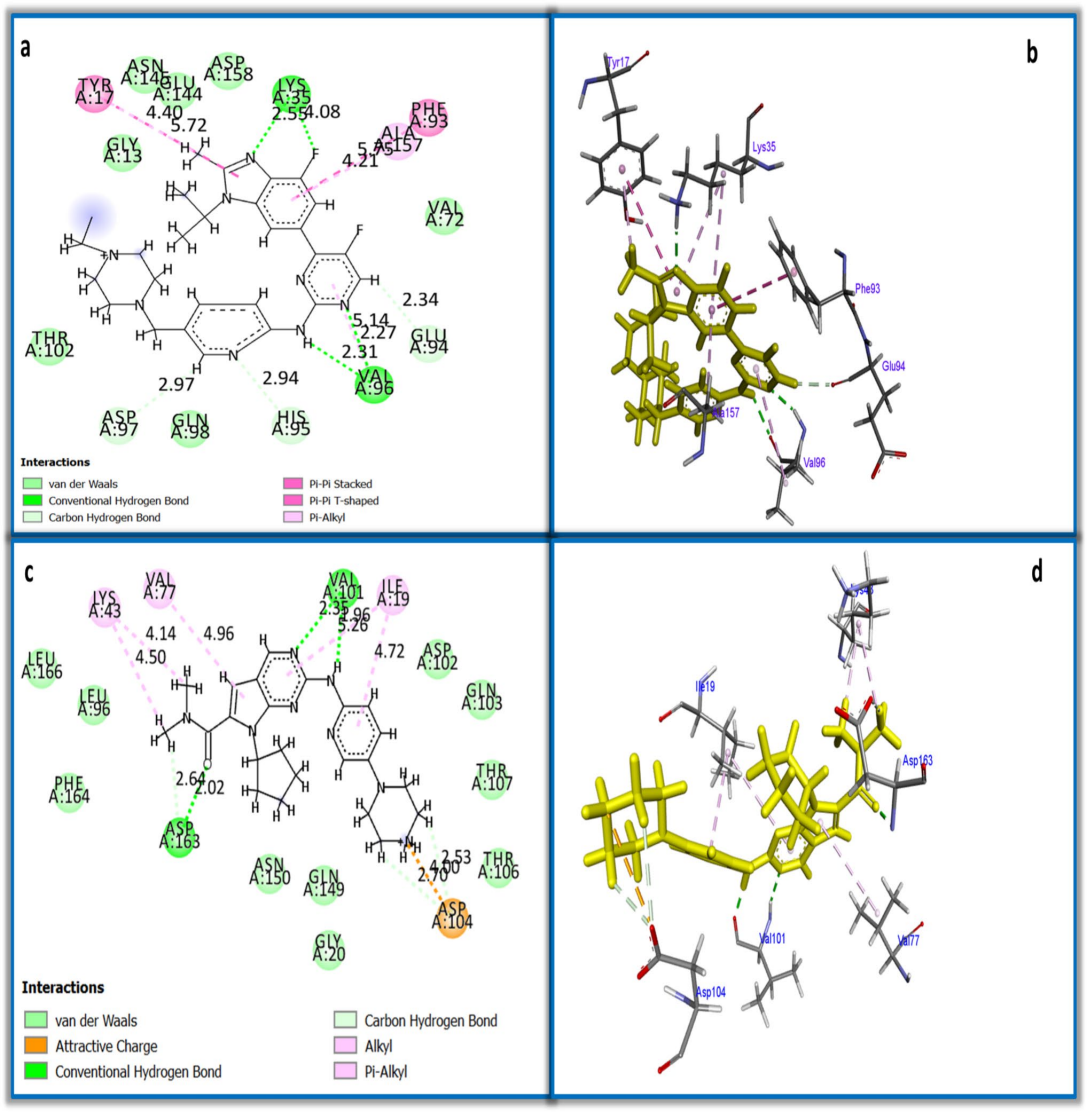
Two and three-dimensional representations of the molecular interaction between **a** and **b** co-crystallized ligand and CDK4, and **c** and **d** co-crystallized ligand and CDK6

The molecular simulation studies were done to detect the possible molecular binding of compound **5b** with the active domains of CDK4 and CDK6. The root mean squared deviation (RMSD) was 1.19 and 0.85 for CDK4 and CDK6, respectively, demonstrating the high accuracy of the results. It was found that compound **5b** interacted with CDK4 with good binding energy (− 26.7 kcal/mol), compared to its co-crystallized ligand (− 36.3 kcal/mol) (Table 2). Compound **5b** bound with the active site of CDK6 with comparable binding energy (− 30.77 kcal/mol) to the standard co-crystallized ligand (− 34.12 kcal/mol) (Table 2). Figure 4a–b showed 12 interactions between compound **5b** and CDK4, which included three hydrogen bonds with ALA: 16, LYS: 35, and SER: 52 residues; three Pi–Pi T-shaped hydrophobic interactions with TYR: 17 and PHE: 93; and six Pi-alkyl hydrophobic interactions with ALA: 33, ALA: 157, VAL: 20, and LEU: 147 residues. It was noticed that compound **5b** interacted with CDK6 with a lower number of interactions than those with CDK4 (six interactions), including two hydrogen bonds with VAL: 10, two Pi-anion electrostatic interactions with ASP: 104, and two hydrophobic interactions with PHE: 98 (Fig. 4c–d). Figure 5a–b shows 12 interactions between the co-crystallized ligand (abemaciclib) and CDK4, including four conventional hydrogen bonds with LYS: 35 and VAL: 96, three carbon hydrogen bonds, and five hydrophobic interactions. Twelve interactions were seen for the co-crystallized ligand (ribociclib) and CDK6, which included three conventional hydrogen bonds, three carbon hydrogen bonds, one attractive charge, and five hydrophobic interactions (Fig. 5c–d). These results supported our results in the qPCR section. Therefore, we could conclude that compound **5b** interacted with the same active sites of CDK4 and CDK6 as the inhibitors abemaciclib and ribociclib, respectively.

**Table 2:** The binding energy values (kcal/mol) and the mode of interactions of compound **5b** and the respective co-crystallized ligands with the active site of CDK4 and CDK6

Protein	Compound 5b			
	**Binding energy (Kcal/mol)**	**Interactions**	**Type of interaction**	**Bond distance** (A^0^)
**CDK4**	** − 26.7**	**O……ALA: 16** **N…….LYS: 35** **Cl…….SER: 52** **Benzene…….TYR: 17** **Benzene……..TYR: 17** **Benzene…….PHE: 93** **Benzene…….ALA: 33** **Benzene…….VAL: 20** **Benzene…….ALA: 157** **Benzene……LEU: 147** **Pyrazole……VAL: 20** **Pyrazole……ALA: 157**	**Conventional hydrogen bond** **Conventional hydrogen bond** **Carbon hydrogen bond** **Pi-Pi T-shaped** **Pi-Pi T-shaped** **Pi-Pi T-shaped** **Pi-alkyl** **Pi-alkyl** **Pi-alkyl** **Pi-alkyl** **Pi-alkyl** **Pi-alkyl**	**2.73** **2.48** **2.96** **5.07** **5.53** **5.6** **4.31** **5.14** **4.72** **4.99** **4.66** **3.91**
**CDK6**	** − 30.77**	**O………VAL: 101** **H………VAL: 101** **Benzene…..ASP: 104** **Benzene…..ASP: 104** **Cl……….PHE: 98** **Benzene…PHE: 98**	**Conventional hydrogen bond** **Carbon hydrogen bond** **Pi-anion** **Pi-anion** **Pi-alkyl** **Pi-Pi T-shaped**	**2.47** **2.53** **4.04** **4.97** **3.66** **5.66**
	**Co-crystallized ligand (Abemaciclib)**			
**CDK4**	** − 36.3**	**Imidazole………..TYR: 17** **Imidazole…………TYR: 17** **N…………………….LYS: 35** **F……………………..LYS: 35** **NH………………….VAL: 96** **N…………………….VAL:96** **Pyrimidine……….VAL:96** **H…………………….GLU:94** **N……………………..HIS: 95** **H……………………..ASP: 97** **Benaene…………..PHE: 93** **Benzene…………..PHE: 93**	**Pi-Pi Stacked** **Pi-alkyl** **Conventional hydrogen bond** **Conventional hydrogen bond** **Conventional hydrogen bond** **Conventional hydrogen bond** **Pi-alkyl** **Carbon hydrogen bond** **Carbon hydrogen bond** **Carbon hydrogen bond** **Pi-Pi T-shaped** **Pi-alkyl**	**5.72** **4.40** **2.55** **4.08** **2.31** **2.27** **5.14** **2.34** **2.94** **2.97** **5.75** **4.21**
	**Co-crystallized ligand (ribociclib)**			
**CDK6**	** − 34.12**	**N…………………..VAL: 101** **NH………………..VAL: 101** **Pyrimidine………ILE: 19** **Pyridine………….ILE: 19** **H……………………ASP: 104** **N…………………….ASP: 104** **H……………………ASP: 104** **O……………………ASP: 163** **H……………………ASP: 163** **Pyrazole…………..VAL: 77** **Methyl…………….LYS: 43** **H…………………….LYS: 43**	**Conventional hydrogen bond** **Conventional hydrogen bond** **Pi-alkyl** **Pi-alkyl** **Carbon hydrogen bond** **Attractive charge** **Carbon hydrogen bond** **Conventional hydrogen bond** **Carbon hydrogen bond** **Pi-alkyl** **Alkyl** **Alkyl**	**2.35** **1.96** **5.26** **4.72** **2.53** **4.00** **2.70** **2.02** **2.64** **4.96** **4.14** **4.50**

## Conclusion

The synthesized chalcone derivatives **5b** and **5c** exhibited the most potent cytotoxic effects against HEP2 and MCF-7 cancer cell lines, respectively. Their anticancer activity appears to be mediated through multiple mechanisms, including G0/G1 and G2/M phase cell cycle arrest, P53-independent apoptosis, autophagy induction independent of ATG3, and ferroptosis, as confirmed by flow cytometric and qPCR analyses in both cell lines. These findings were further supported by molecular docking studies, which reinforced the proposed molecular interactions of compound **5b** with key regulatory targets. However, our study has several limitations that should be acknowledged. First, *CASP8* expression in MCF-7 cells treated with compound **5c** showed relatively high inter-experimental variability, and further studies with additional replicates and/or time-course experiments are required to clarify its precise contribution. Second, the use of a limited number of cell lines was due to financial limitations. Finally, there is a lack of in vivo validation and toxicity evaluation. So, future research should concentrate on evaluating the pharmacokinetic and pharmacodynamic characteristics of the compounds, encompassing their absorption, distribution, metabolism, and excretion (ADME) profiles, in addition to their potential toxicity and adverse effects. The results of these in vivo studies will be essential for assessing the therapeutic potential of the compounds and guiding their subsequent development as potential therapeutic agents. However, the present findings highlight **5b** and **5c** as promising dual CDK inhibitors and apoptosis inducers, warranting further preclinical evaluation as lead anticancer candidates.

## Material and methods

### Chemistry

“Melting points were measured using a Stuart melting point apparatus and were uncorrected. The IR spectra were recorded using an FTIR Bruker-vector 22 spectrophotometer as KBr pellets. The ^1^H and ^13^C NMR spectra were recorded in DMSO-*d*_*6*_ as a solvent with a Varian Mercury VXR-300 NMR spectrometer operating at 300 MHz and 75 MHz, using TMS as an internal standard. Chemical shifts were reported as *δ* values in ppm. Mass spectra were recorded with a Shimadzu GCMS-QP-1000 EX mass spectrometer in EI (70 eV) model. The elemental analyses were performed at the Micro Analytical Centre, Cairo University.”

#### General procedure for the synthesis of compounds 5a–5d

A mixture of cyanoacetyl-indole **4** (1 mmol) and appropriate aldehyde **3a–d** (1 mmol) was heated at reflux in absolute EtOH (10 mL) containing piperidine (0.2 mL) for 30 min. The crude product was collected and crystallized from the EtOH/dioxane mixture.

#### (*E*)−2-(4-(2-Cyano-3-(1*H*-indol-3-yl)−3-oxoprop-1-en-1-yl)phenoxy)-*N*-phenylacetamide (5a)


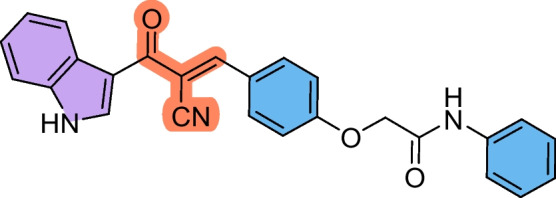


Pale yellow crystals (88%); M.p: 276–278.

IR (KBr): ν_max_/cm^−1^ 3215 (NH), 2242 (CN), 1720 (CO).^1^H NMR (500 MHz, DMSO-*D*_6_) δ 4.85 (s, 2H, CH2), 7.06 (t, 1H, Ar–H, J = 5 Hz), 7.19 (d, 2H, Ar–H, *J* = 5 Hz), 7.24 (t, 2H, Ar–H, *J* = 5 Hz), 7.30 (t, 2H, Ar–H, *J* = 5 Hz), 7.52 (d, 1H, Ar–H, *J* = 5 Hz), 7.62 (d, 2H, Ar–H, *J* = 10 Hz), 8.07 (d, 2H, Ar–H, *J* = 5 Hz), 8.17 (m, 2H, Ar–H + vinyl-H), 8.43 (s, 1H, indole-H2), 10.17 (br. s, 1H, NH), 12.24 (br. s, 1H, NH). ^13^C NMR (126 MHz, DMSO-D6) δ 67.7, 109.0, 113.0, 114.3, 116.0, 118.9, 120.3, 122.0, 122.9, 124.0, 124.3, 126.1, 126.8, 129.3, 133.3, 136.0, 137.2, 138.9, 152.4, 161.8, 166.5, 182.0. MS (EI, 70 eV): 421 [M^+^], Anal. Calcd. for C_26_H_19_N_3_O_3_: C, 74.10; H, 4.54; N, 9.97. Found: C, 74.24; H, 4.67; N, 9.83.

#### (E)-*N*-(4-Chlorophenyl)−2-(4-(2-cyano-3-(1*H*-indol-3-yl)−3-oxoprop-1-en-1-yl)phenoxy)acetamide (5b)


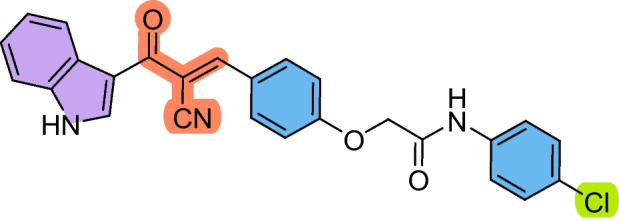


Pale yellow crystals (82%); M.p: 290–294.

IR (KBr): ν_max_/cm^−1^ 3222 (NH), 2240 (CN), 1715 (CO). ^1^H NMR (500 MHz, DMSO-*D*_6_) δ 4.84 (s, 2H, CH_2_), 7.18–7.24 (m, 4H, Ar–H), 7.36 (d, 2H, Ar–H, *J* = 10 Hz), 7.51 (d, 1H, Ar–H, *J* = 10 Hz), 7.64 (d, 2H, Ar–H, J = 10 Hz), 8.05 (d, 2H, Ar–H, *J* = 10 Hz), 8.18 (br, 2H, Ar–H + vinyl-H), 8.41 (s, 1H, indole-H2), 10.31 (br. s, 1H, NH), 12.22 (br. s, 1H, NH). ^13^C NMR (126 MHz, DMSO-D6) δ 67.7, 109.1, 113.0, 114.3, 116.0, 118.8, 121.8, 122.0, 122.9, 124.0, 126.1, 126.8, 127.9, 129.2, 133.2, 136.0, 137.2, 137.9, 152.3, 161.7, 166.7, 182.0. MS (EI, 70 eV): 455 [M^+^], Anal. Calcd. for C_26_H_18_ClN_3_O_3_: C, 68.50; H, 3.98; N, 9.22. Found: C, 68.63; H, 4.11; N, 9.33.

#### (*E*)−2-(4-(2-Cyano-3-(1*H*-indol-3-yl)−3-oxoprop-1-en-1-yl)phenoxy)-*N*-(*p*-tolyl)acetamide (5c)


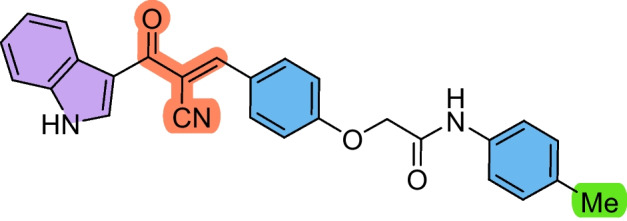


Pale yellow crystals (87%); M.p: 271–273.

IR (KBr): ν_max_/cm^−1^ 3222 (NH), 2220 (CN), 1720 (CO).^1^H NMR (500 MHz, DMSO-*D*_6_) δ 2.22 (s, 3H, CH_3_), 4.81 (s, 2H, CH_2_), 7.09 (d, 2H, Ar–H, *J* = 10 Hz), 7.17 (d, 2H, Ar–H, *J* = 10 Hz), 7.24 (m, 2H, Ar–H), 7.50 (m, 3H, Ar–H), 8.05 (d, 2H, *J* = 10 Hz), 8.14 (d, 1H, Ar–H, *J* = 10 Hz), 8.17 (s, 1H, vinyl-H), 8.41 (s, 1H, indole-H2), 10.07 (br. s, 1H, NH), 12.23 (br. s, 1H, NH). ^13^C NMR (126 MHz, DMSO-D6) δ 21.0, 39.7, 39.8, 40.0, 40.2, 40.3, 40.5, 40.7, 67.8, 109.0, 113.0, 114.3, 116.0, 118.8, 120.3, 122.0, 122.8, 124.0, 126.1, 126.8, 129.7, 133.2, 135.9, 136.4, 137.2, 152.3, 161.8, 166.2, 181.9. MS (EI, 70 eV): 435 [M^+^], Anal. Calcd. for C_27_H_21_N_3_O_3_: C, 74.47; H, 4.86; N, 9.65. Found: C, 74.52; H, 4.94; N, 9.77.

#### (*E*)−2-(4-(2-Cyano-3-(1*H*-indol-3-yl)−3-oxoprop-1-en-1-yl)phenoxy)-*N*-(4-methoxyphenyl)acetamide (5d)


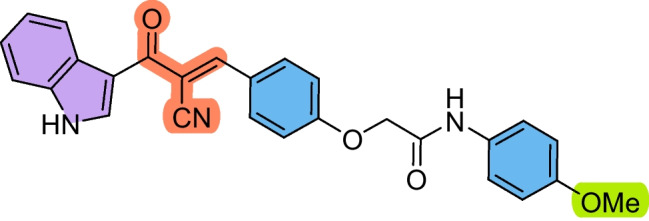


Pale yellow crystals (78%); M.p: 267–269.

IR (KBr): ν_max_/cm^−1^ 3220 (NH), 2222 (CN), 1718 (CO).^1^H NMR (500 MHz, DMSO-*D*_6_) δ 3.70 (s, 3H, OCH_3_), 4.80 (s, 2H, CH_2_), 6.87 (d, 2H, Ar–H, *J* = 10 Hz), 7.18 (d, 2H, Ar–H, *J* = 10 Hz), 7.23–7.27 (m, 2H, Ar–H), 7.51–7.52 (m, 2H, Ar–H), 8.14 (d, 1H, Ar–H, *J* = 10 Hz), 8.18 (s, 1H, vinyl-H), 8.41 (s, 1H, indole-H2), 10.02 (br. s, 1H, NH), 12.23 (br. s, 1H, NH). ^13^C NMR (126 MHz, DMSO-D6) δ 39.7, 39.8, 40.0, 40.2, 40.3, 40.5, 40.7, 55.8, 67.8, 109.0, 113.0, 114.3, 114.5, 116.0, 118.8, 122.0, 122.8, 124.0, 126.1, 126.8, 131.9, 133.2, 135.9, 137.2, 152.3, 156.2, 161.8, 166.0, 182.0.. MS (EI, 70 eV): 451 [M^+^], Anal. Calcd. for C_27_H_21_N_3_O_4_: C, 71.83; H, 4.69; N, 9.31. Found: C, 71.96; H, 4.78; N, 9.45.

### Biological assays

#### Cell culture

The human cancer cell lines used in this study included lung carcinoma (A549), breast cancer (MCF-7), and laryngeal carcinoma (HEP2), along with the normal human fibroblast cell line WI-38. A549 and MCF-7 cells were cultured in RPMI-1640 medium (Gibco, Thermo Fisher Scientific, USA), while HEP2 and WI-38 cells were maintained in Dulbecco’s Modified Eagle Medium (DMEM; Gibco). All culture media were supplemented with 10% fetal bovine serum (FBS; Gibco), 1% L-glutamine, and 1% penicillin–streptomycin (Gibco). Cells were grown at 37 °C in a humidified incubator with 5% CO_2_. The media were refreshed every 2–3 days, and cells were sub-cultured upon reaching approximately 80% confluency using 0.5% trypsin–EDTA (Gibco). The cell viability was routinely checked using Trypan blue exclusion (Catalog# 15,250,061) (Gibco). All cell lines were regularly tested for mycoplasma using MycoFluor™ Mycoplasma Detection Kit (Catalog# M7006).

#### MTT assay

The MTT assay was performed to evaluate the cytotoxic effect of novel chalcone series **5a–d** on different cell lines, namely, A549, MCF-7, HEP2, and normal cell line WI-38. Cells were seeded in 96-well plates at a concentration of 1 × 10^4^ cells per well and allowed to adhere overnight. After 24 h, cells were treated with a serial range of concentrations of chalcone compounds **5a–d** (0.1–200 µg/mL) diluted in serum-free culture medium. Each concentration was added in triplicate. Dimethyl sulfoxide (DMSO) was used as a vehicle to solubilize the compounds. The final concentration of DMSO did not exceed 0.1% (v/v). 5-FU was used as a positive control. For the negative control, cells were incubated with serum-free culture medium containing the same final concentration of DMSO (vehicle) as in the treated wells but without chalcone, and this condition was taken as 100% viability. Following a 48-h incubation period, the culture medium was removed, and 100 µL of fresh medium containing 0.5 mg/mL of MTT reagent (3-(4,5-dimethylthiazol-2-yl)−2,5-diphenyltetrazolium bromide, Sigma-Aldrich, St. Louis, MO, USA) was added to each well. Cells were then incubated for an additional 4 h at 37 °C in a humidified incubator with 5% CO_2_. After incubation, the MTT solution was carefully removed, and 150 µL of DMSO (Sigma-Aldrich) was added to each well to solubilize the formazan crystals formed by metabolically active cells. The absorbance of the resulting solution was measured at 570 nm using a microplate reader (Bio-Rad, Hercules, CA, USA). The absorbance readings were directly proportional to the number of viable cells. IC₅₀ values (the concentration of chalcone required to inhibit cell growth by 50%) were calculated by fitting concentration–response curves using nonlinear regression log(concentration) vs. response GraphPad Prism software (version 8.0, GraphPad Software, San Diego, CA, USA).

#### Flow cytometry

##### Autophagy assay

Autophagic vacuoles were stained using CYTO-ID® Green detection reagent (fluorescent marker; CYTO-ID® Autophagy Detection Kit, Enzo Life Sciences, ENZ-51031), a cationic amphiphilic dye that selectively labels autophagic vesicles (pre-autophagosomes, autophagosomes, and autolysosomes) in live cells. HEP2 and MCF-7 cells were seeded and treated for 48 h with compound **5b** or **5c**, respectively, at their IC₅₀ concentrations. After treatment, cells were harvested, washed twice with 1 × assay buffer supplied with the kit, and resuspended in staining solution containing CYTO-ID Green detection reagent diluted in 1 × assay buffer according to the manufacturer’s instructions. Cell suspensions were incubated for 30 min at 37 °C in the dark, washed once with 1 × assay buffer, and immediately analyzed by flow cytometry using a 488-nm excitation laser and the FITC (FL1) channel for emission detection. Autophagy levels were expressed as the fold increase in mean fluorescence intensity in treated samples relative to the corresponding vehicle-treated control (Guo et al. 2015).

##### Cell cycle analysis

To analyze the effects of **5b** and **5c** on cell cycle distribution, propidium iodide (PI) staining was performed (Michalkova et al. 2022). Cells were fixed in 70% ethanol overnight at − 20 °C after 48 h of treatment with IC_50_ concentrations of **5b** and **5c**. The fixed cells were washed and incubated with 50 µg/mL PI and 100 µg/mL RNase A for 30 min at 37 °C in the dark. Cell cycle analysis was performed using a BD FACSCalibur flow cytometer, and the distribution of cells in G0/G1, S, and G2/M phases was analyzed using FlowJo software.

##### Apoptosis assay

Apoptosis was assessed using Annexin V-FITC and propidium iodide (PI) staining to differentiate between live, early apoptotic, and late apoptotic/necrotic cells. HEP2 and MCF-7 cells were seeded in 12-well plates at a density of 6–8 × 10^5^ cells per well and allowed to adhere for 24 h. Cells were treated with IC_50_ concentrations of **5b** for HEP2 cells and **5c** for MCF-7 cells for 48 h. Untreated cells were used as the negative control. After incubation, the cells were harvested by trypsinization and centrifuged at 1200 rpm for 10 min at 4 °C. After discarding the supernatant, the cells were washed with phosphate-buffered saline (PBS) and resuspended in a binding buffer. The cells were then stained with Annexin V-FITC and PI according to the manufacturer’s instructions (BD Biosciences). The samples were analyzed using a BD FACSCalibur flow cytometer, and data analysis was performed using FlowJo software.

#### Quantitative real-time PCR

The expression level of the following ten genes (*P53*,* BAX*, *Bcl2*, *Caspase-8*,* LC3B*,* ATG3*,* HO*,* TF*, *CDK4*, and *CDK6*) was examined after 48 h of treatment of HEP2 and MCF7 with IC_50_ values of **5b** and **5c**, respectively, using real-time polymerase chain reaction (qPCR). Total RNA was extracted from cultured cells utilizing the GeneJET RNA Purification Kit (Thermo Fisher Scientific, USA). Subsequently, complementary DNA (cDNA) synthesis was performed using 1 µg of RNA with the cDNA Reverse Transcription Kit (Applied Biosystems, Foster City, CA, USA). Quantification of target gene expression was conducted through real-time polymerase chain reaction (qPCR) employing the SYBR™ Green PCR Master Mix (Applied Biosystems, USA) on the StepOnePlus Real-Time PCR System (Applied Biosystems), using the protocol cycles condition including an initial denaturation step at 95 °C for 10 min, followed by 40 cycles of denaturation at 95 °C for 15 s, and annealing and extension at 60 °C for 1 min. The relative gene expression levels were calculated based on the 2⁻ΔΔCT method (Livak and Schmittgen 2001). The primer sequences were utilized as we described before (Emad et al. 2023). Values were presented as relative expression levels and normalized to 18S rRNA.

#### Molecular docking

The Molecular Operating Environment (MOE) version 2009.10 was used to perform the molecular simulation studies (Sroor et al. 2023). The target compound **5b** was drawn using the builder interface, and the local energy minimization was determined using MOPAC. Afterward, the global energy minimization was estimated using the conformational search, where RMS distance and RMS gradient were set at 0.1 Å and 0.01 kcal/mol, respectively. The X-ray crystallographic structure of CDK4 and CDK6 proteins complexed with their standard co-crystallized ligands (PDB ID: 7SJ3 and 5L2T), respectively, was gained from the protein databank. Several modifications were made on the selected proteins as follows: firstly, the protonation of the whole protein was carried out. Afterward, the unwanted water chains and self-ligands were removed. After that, the MOE alpha site finder was used to determine the selected proteins’ active domains. Finally, after the self-docking step of the altered protein with its co-crystallized ligand, it was then exposed to the target compounds to reveal the protein-compound interactions at the active site. The protein-compound interactions were visualized in two-dimensional and three-dimensional forms using BIOVIA Discovery Studio V6.1.0.15350.

#### Statistical analysis

All the experiments were performed in triplicate and repeated at least three times. IC_50_ values were calculated using nonlinear regression (log concentration vs. response) GraphPad Prism software (version 8.0, GraphPad Software, San Diego, CA, USA), where IC_50_ was presented as means ± SD. Statistical significance was evaluated using Student’s t-test, and the values were considered significant at *P* < 0.05. The selectivity index of the synthesized compounds was calculated as SI = IC_50_ of the target compound in a normal cell line/IC_50_ of the same compound in the cancer cell line.

## Supplementary Information

Below is the link to the electronic supplementary material.

ESM1 (DOCX 988 KB)

## Data Availability

All data generated or analyzed during this study are included in this published article and its supplementary information file. Data available at request (Ismail A. Abdelhamid, ismail_shafy@yahoo.com, ismail_shafy@cu.edu.eg).
