# Supplementary material for: Synthesis and cytotoxicity of novel cyanochalcones: induction of cell cycle arrest, apoptosis, and autophagy in HEP2 and MCF-7 cells
Source: Naunyn Schmiedebergs Arch Pharmacol. 2026 Jan 15;399(6):8867–82. doi: 10.1007/s00210-025-04940-z (PMC13086721; doi:10.1007/s00210-025-04940-z)
Supplement: Supplementary file 1 — (DOCX 988 KB) [file 210_2025_4940_MOESM1_ESM.docx]

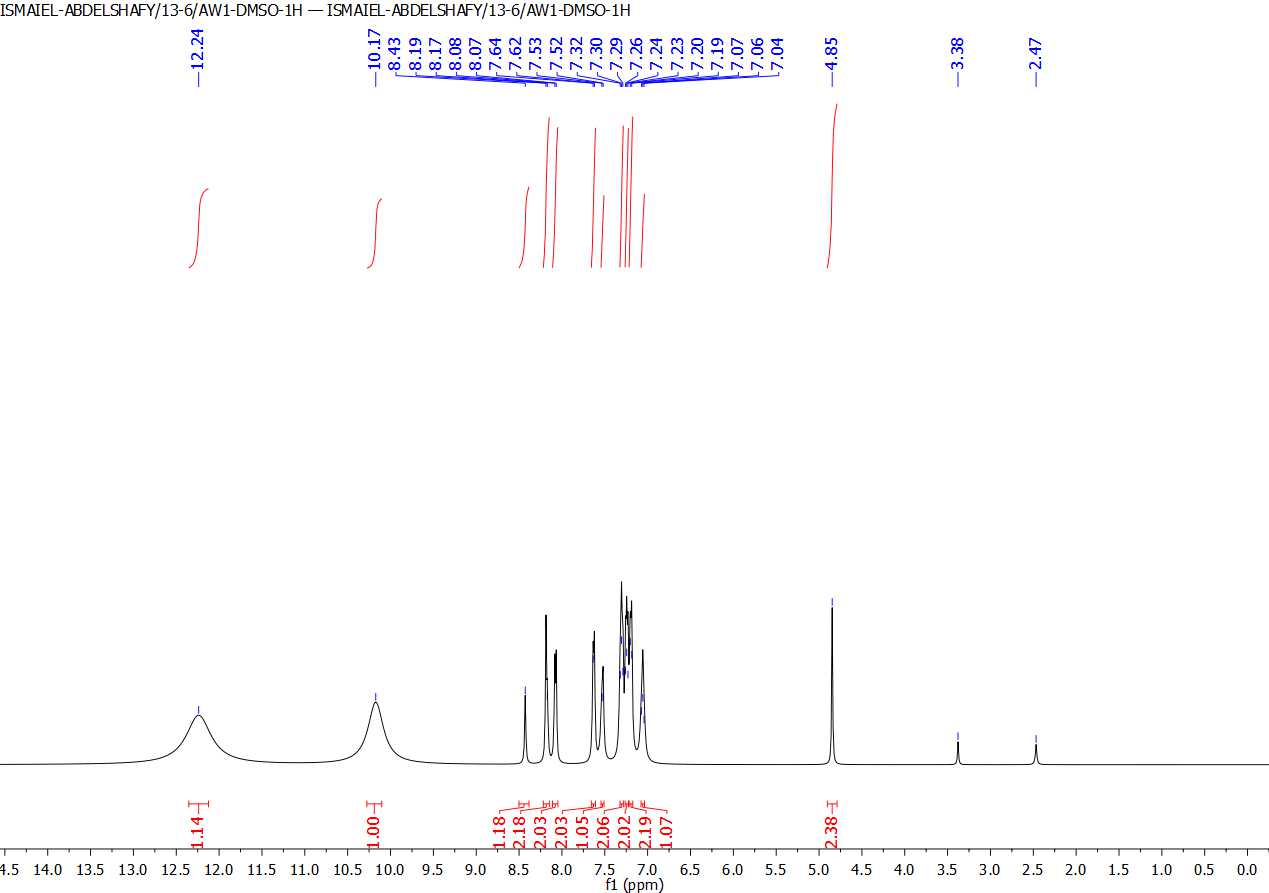

**Figure S1**. ^1^H NMR of compound **5a**


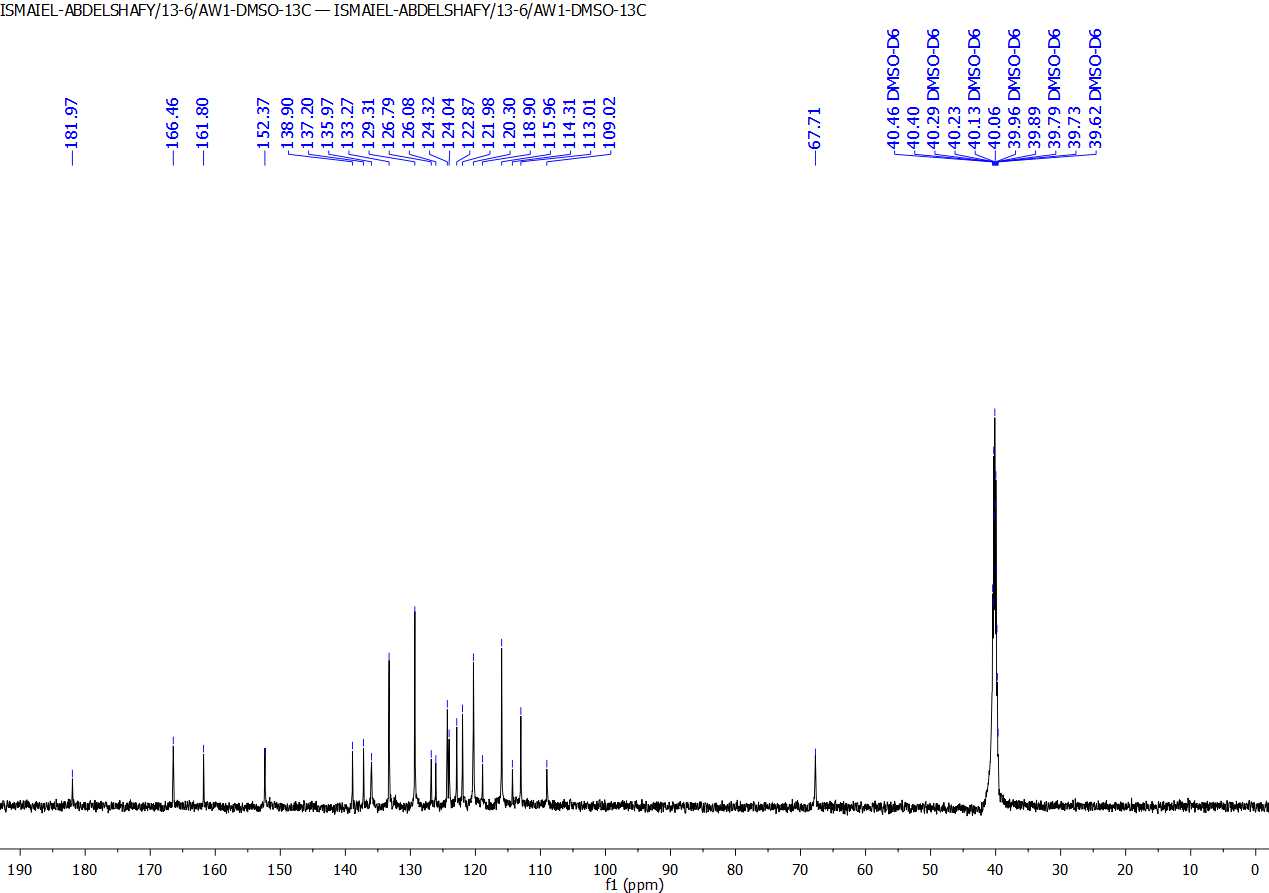

**Figure S2**. ^13^C NMR of compound **5a**


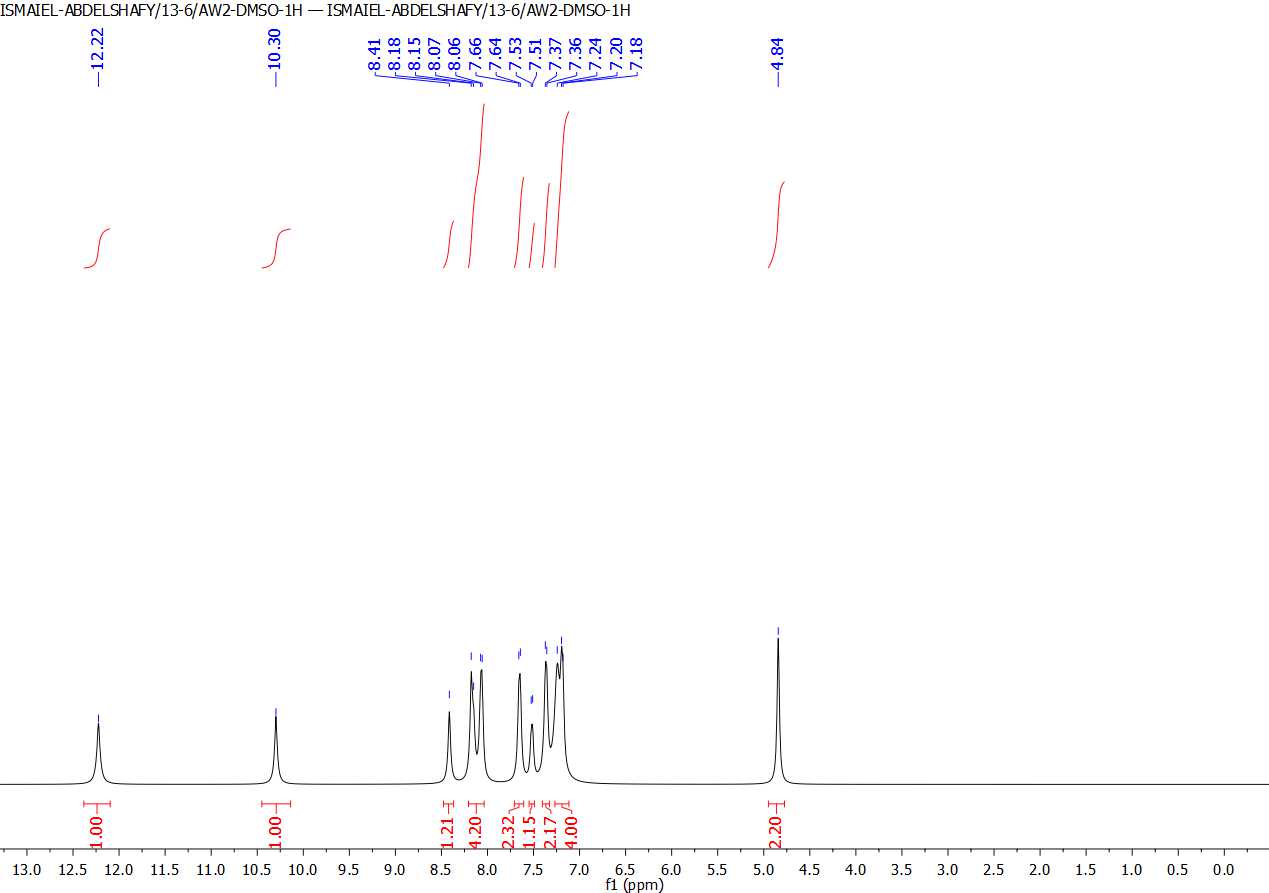

**Figure S3**. ^1^H NMR of compound **5b**


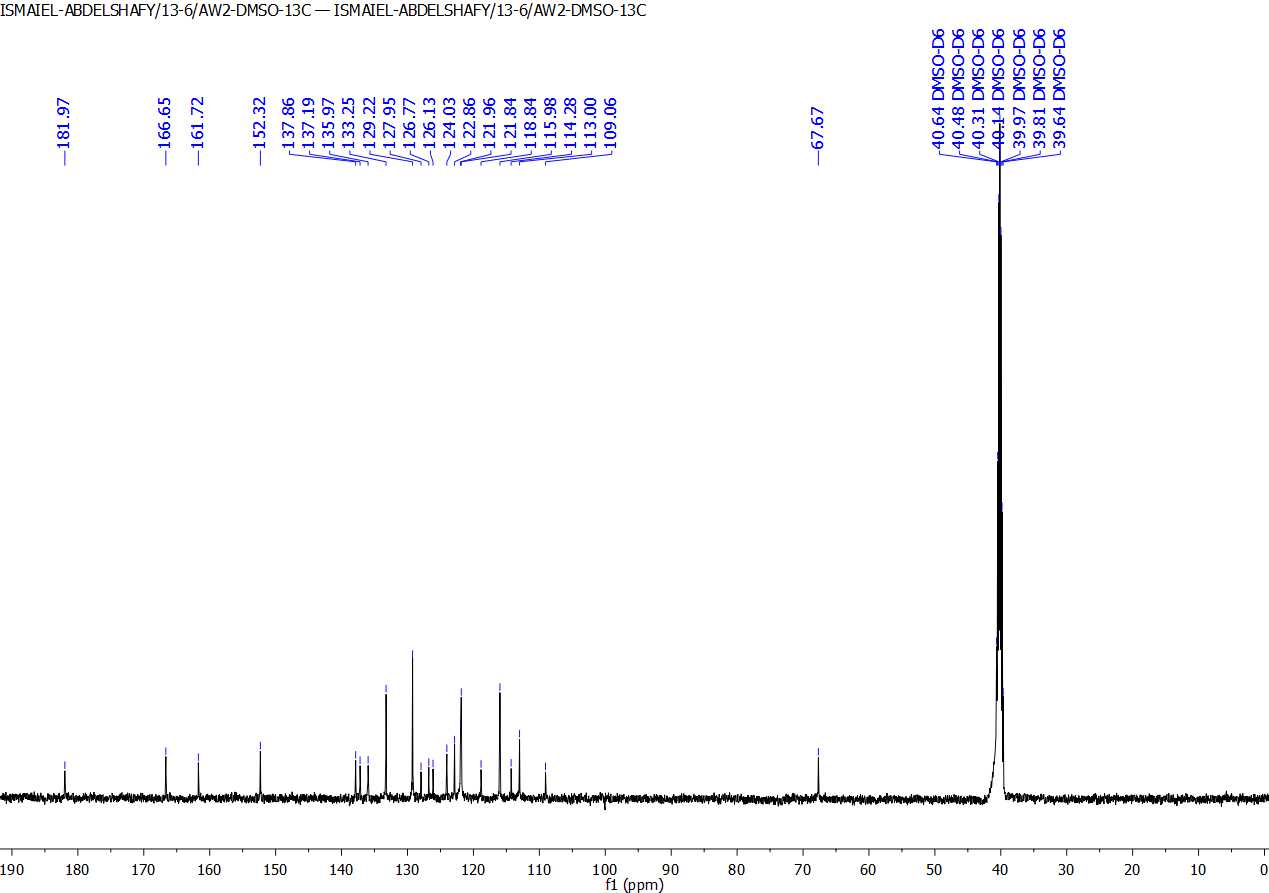

**Figure S4.** ^13^C NMR of compound **5b**


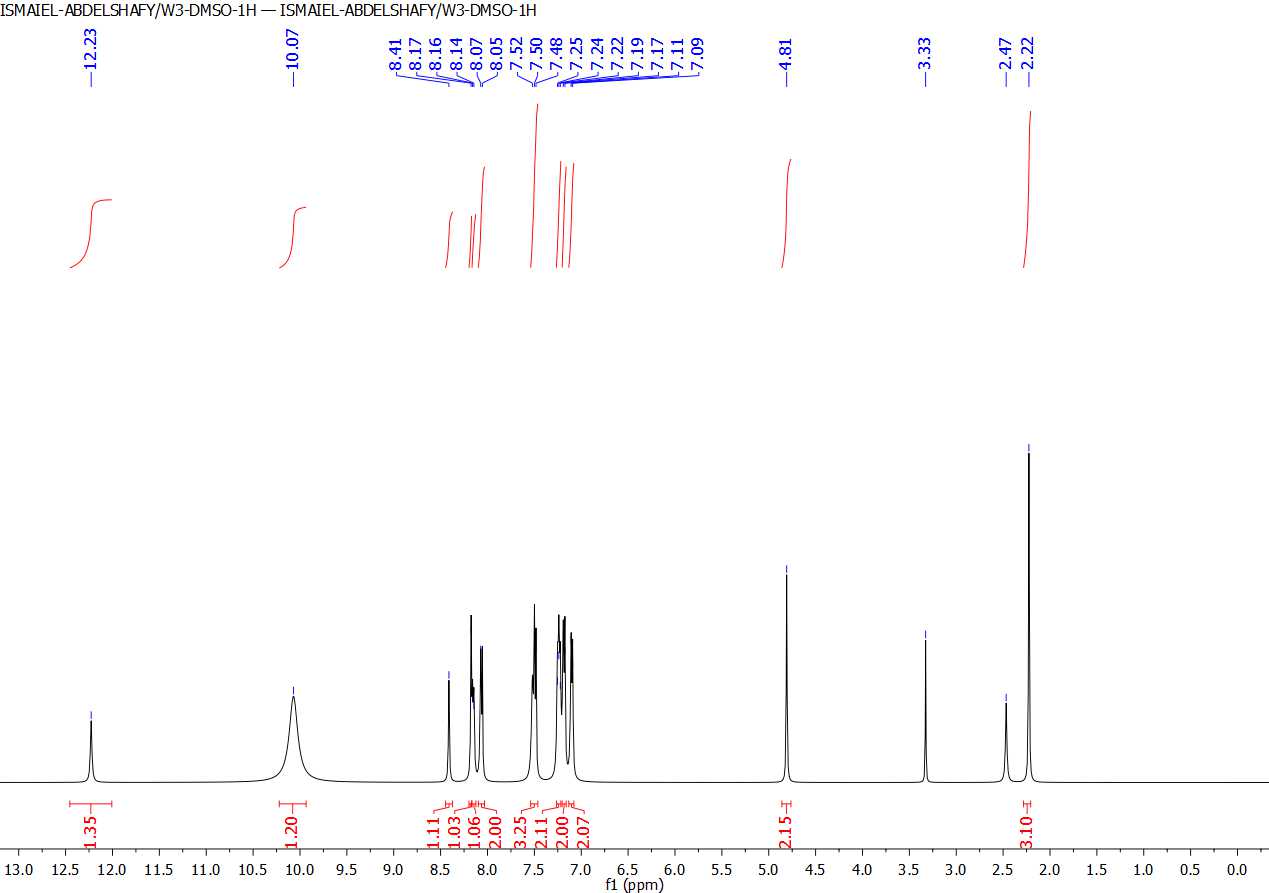

**Figure S5**. ^1^H NMR of compound **5c**


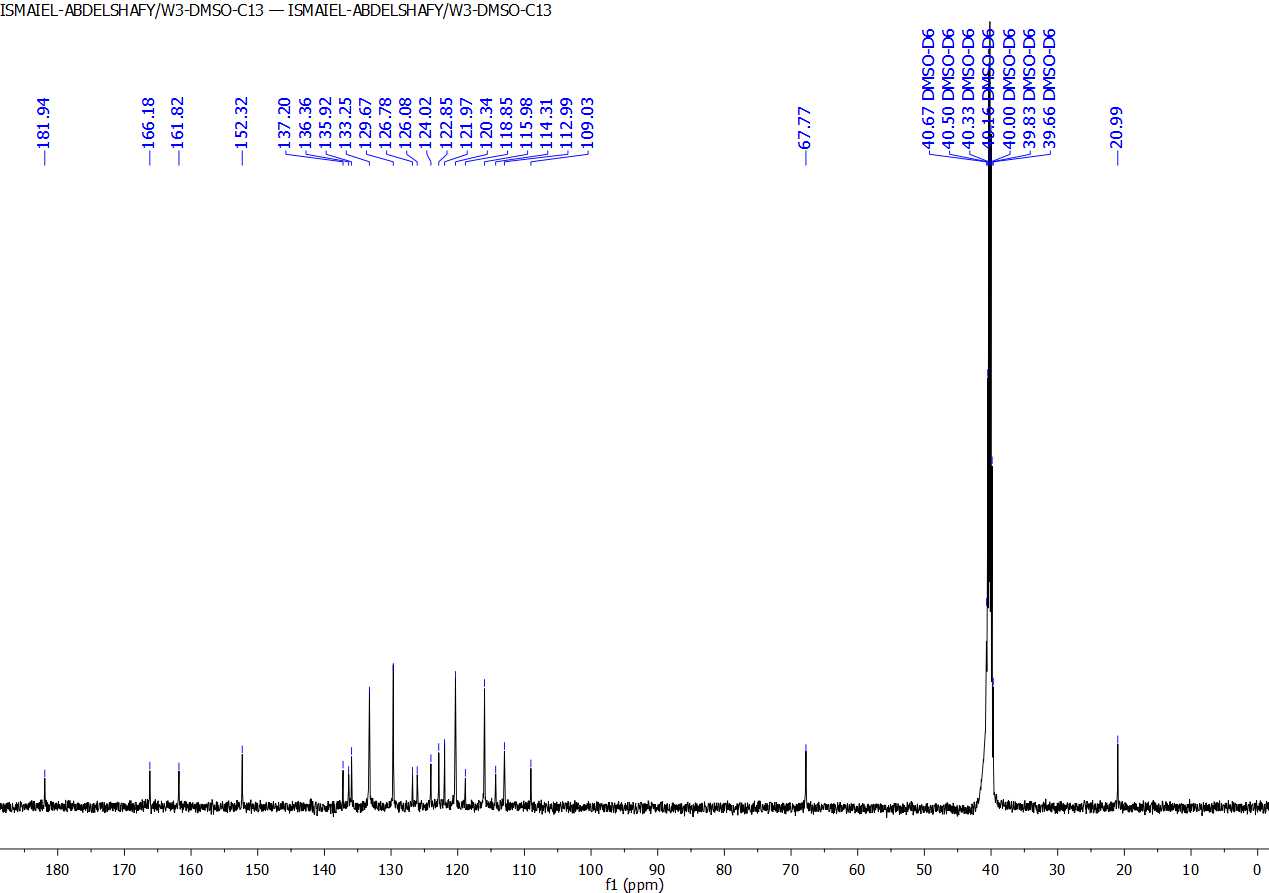

**Figure S6**. ^13^C NMR of compound **5c**


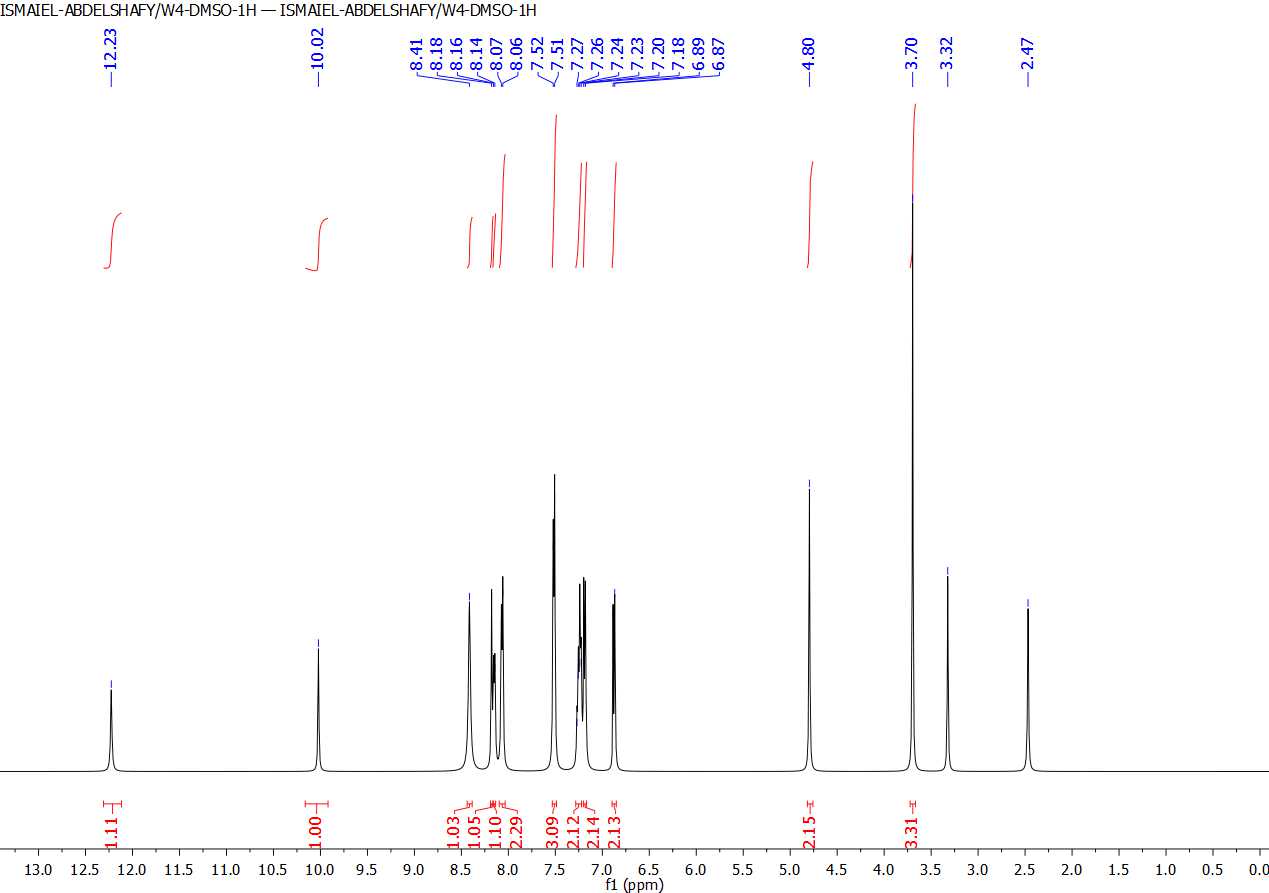

**Figure S7**. ^1^H NMR of compound **5d**


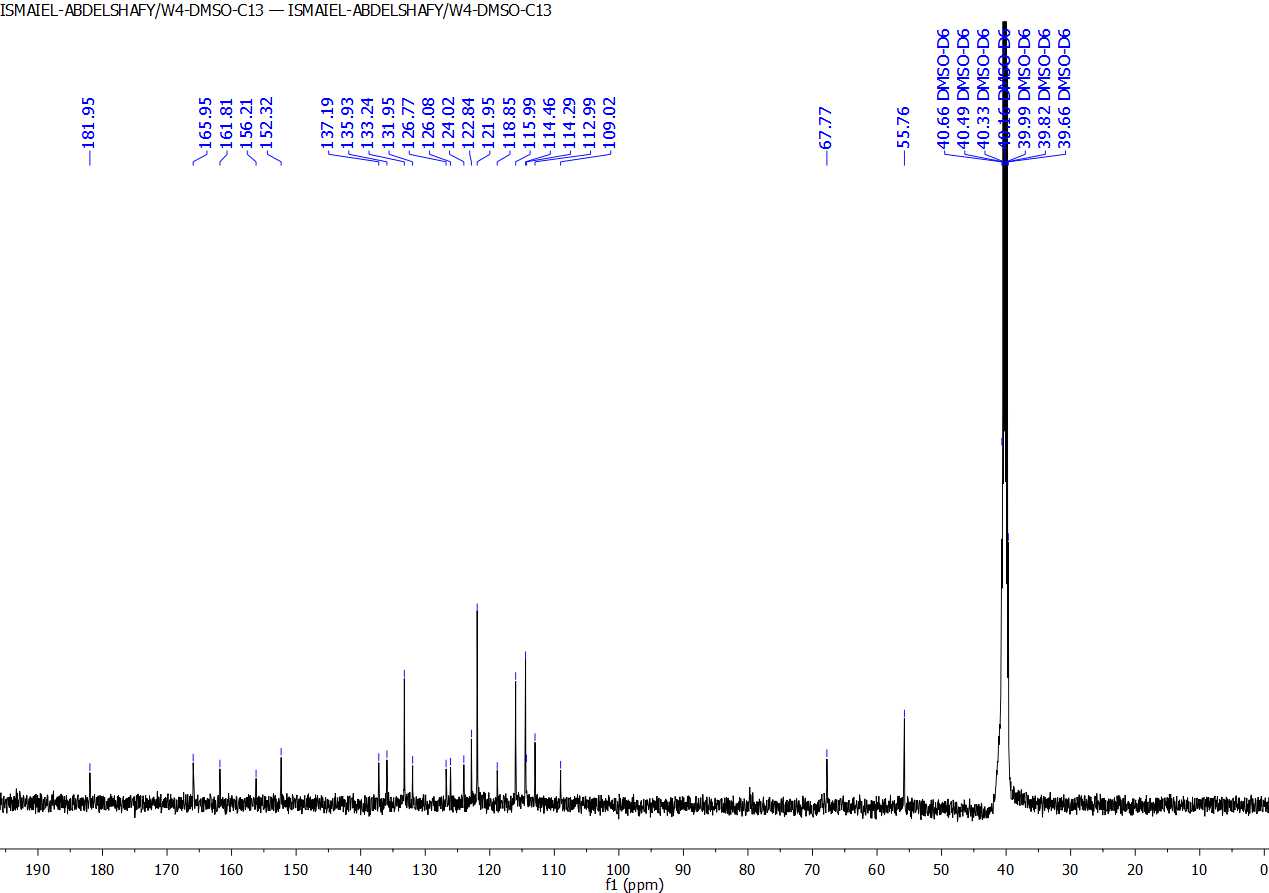

**Figure S8.** ^13^C NMR of compound **5d**

**
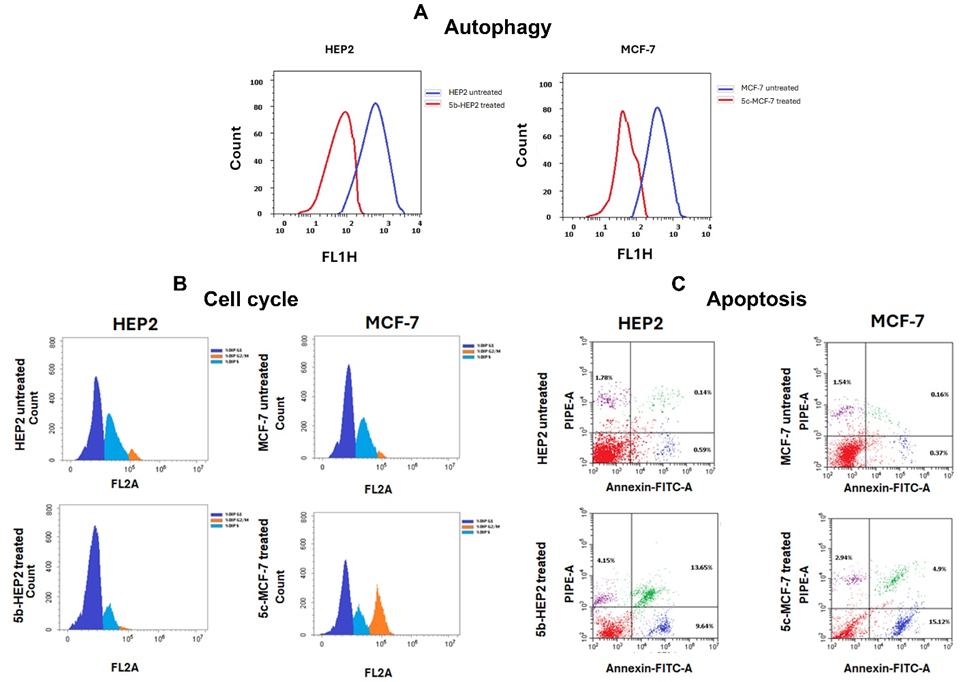
**

**Figure S9.** Figure 2. Effects of chalcone derivatives **5b** and **5c** on autophagy, cell cycle progression, and apoptosis in HEP2 and MCF-7 cells. HEP2 cells were treated with compound **5b** (23.93 µM, IC₅₀) and MCF-7 cells were treated with compound **5c** (39.08 µM, IC₅₀) for 48 h; untreated cells served as negative controls. (A) Representative flow-cytometric histograms of CYTO-ID^®^ Green fluorescence showing autophagic vacuoles in untreated and treated HEP2 and MCF-7 cells. (B) Representative DNA-content histograms (PI staining) illustrating cell-cycle distribution in untreated and treated HEP2 and MCF-7 cells. (C) Representative Annexin V-FITC/PI dot plots showing viable, early apoptotic, late apoptotic, and necrotic populations in untreated and treated HEP2 and MCF-7 cells. The lower-left quadrant (Q3; Annexin V⁻/PI⁻) corresponds to viable cells, the lower-right quadrant (Q4; Annexin V⁺/PI⁻) to early apoptotic cells, the upper-right quadrant (Q2; Annexin V⁺/PI⁺) to late apoptotic/secondary necrotic cells, and the upper-left quadrant (Q1; Annexin V⁻/PI⁺) to primary necrotic cells.
